# Does the intraoperative 3D-flat panel control of the planned implant position lead to an optimization and increased in safety in the anatomically demanding region C1/2?

**DOI:** 10.1186/s12893-023-01934-7

**Published:** 2023-02-18

**Authors:** J.-S. Jarvers, U. A. J. Spiegl, P. Pieroh, N. von der Höh, A. Völker, C. Pfeifle, S. Glasmacher, C. E. Heyde

**Affiliations:** Department of Orthopedic Surgery, Traumatology and Plastic Surgery, Leipzig University, Liebigstraße 20, 04103 Leipzig, Germany

**Keywords:** Intraoperative 3D-scan, Intraoperative control, C1/2-instability, Image quality

## Abstract

**Background:**

The aim of this study was to evaluate the applicability and advantages of intraoperative imaging using a 3D flat panel in the treatment of C1/2 instabilities.

**Materials:**

Prospective single-centered study including surgeries at the upper cervical spine between 06/2016 and 12/2018. Intraoperatively thin K-wires were placed under 2D fluoroscopic control. Then an intraoperative 3D-scan was carried out. The image quality was assessed based on a numeric analogue scale (NAS) from 0 to 10 (0 = worst quality, 10 = perfect quality) and the time for the 3D-scan was measured. Additionally, the wire positions were evaluated regarding malpositions.

**Results:**

A total of 58 patients were included (33f, 25 m, average age 75.2 years, r.:18–95) with pathologies of C2: 45 type II fractures according to Anderson/D'Alonzo with or without arthrosis of C1/2, 2 Unhappy triad of C1/2 (Odontoid fracture Type II, anterior or posterior C1 arch-fracture, Arthrosis C1/2) 4 pathological fractures, 3 pseudarthroses, 3 instabilities of C1/2 because of rheumatoid arthritis, 1 C2 arch fracture). 36 patients were treated from anterior [29 AOTAF (combined anterior odontoid and transarticular C1/2 screw fixation), 6 lag screws, 1 cement augmented lag screw] and 22 patients from posterior (regarding to Goel/Harms). The median image quality was 8.2 (r.: 6–10). In 41 patients (70.7%) the image quality was 8 or higher and in none of the patients below 6. All of those 17 patients the image quality below 8 (NAS 7 = 16; 27.6%, NAS 6 = 1, 1.7%), had dental implants. A total of 148 wires were analyzed. 133 (89.9%) showed a correct positioning. In the other 15 (10.1%) cases a repositioning had to be done (n = 8; 5.4%) or it had to be drawn back (n = 7; 4.7%). A repositioning was possible in all cases. The implementation of an intraoperative 3D-Scan took an average of 267 s (r.: 232-310 s). No technical problems occurred.

**Conclusion:**

Intraoperative 3D imaging in the upper cervical spine is fast and easy to perform with sufficient image quality in all patients. Potential malposition of the primary screw canal can be detected by initial wire positioning before the Scan. The intraoperative correction was possible in all patients.

*Trial registration* German Trials Register (Registered 10 August 2021, DRKS00026644—Trial registration: German Trials Register (Registered 10 August 2021, DRKS00026644—https://www.drks.de/drks_web/navigate.do?navigationId=trial.HTML&TRIAL_ID=DRKS00026644)

## Background

The operative therapy of fractures or other instabilities of the upper cervical spine remains a challenging procedure. Minimal implant-misplacement can cause neurovascular complications or material-loosening as a consequence of multiple drilling procedures [1]. Several procedures for the treatment of upper cervical instabilities have been described such as anterior approaches using combined anterior screw osteosynthesis and anterior transarticular C1/2 screw fixation (AOTAF) as well as posterior procedures according to the technique described by Goel/Harms or transarticular C1/2-stabilization regarding to Magerl [2–9].

All methods have in common that screw placement has to be performed in a technical demanding region with small corridor of safety. Traditionally, screw placement is done using intraoperative anatomic landmarks and fluoroscopic control. Intraoperative fluoroscopy is carried out two-dimensionally, i.e. in two planes. However, newer and more innovative C-arms enable the possibility of generation of intraoperative 3D images. The advantages of this technique in spine surgery have been pointed out in several studies dealing with navigated procedures or intraoperative 3D-control alone [10–13]. In this study we aimed to check the envisaged implant position by controlling the drill canal before the screw was actually implanted. Furthermore, the aim of this study was to evaluate the applicability and advantages of intraoperative imaging using a 3D flat panel in the treatment of C1/2 instabilities. We hypothesized that the technique would be easy and provide sufficient image quality improving patient safety.

## Methods

### Patient collective

It is a prospective single center study at a level 1 spine center. This study was approved by the local institutional ethics committee of the medical faculty Leipzig (reference 042/21-ek).

The inclusion criteria were patients who were at least 18 years old with instabilities of the C1/2 region caused by fractures, pseudarthroses, malignancies or rheumatoid arthroses. According to our treatment protocol odontoid fractures type II according to Anderson/D'Alonzo with a typical fracture line (Eysel/Roosen Type A and B) were treated from anterior with one or two lag screw(s). Where there were combined fractures of the odontoid process with or without an anterior or posterior fracture of the C1-arch and an atlanto-odontoid osteoarthritis (AO), AOTAF was performed with one or two odontoid lag screws in addition. In case of a high riding vertebral artery and odontoid fractures with a fracture orientation from anteroinferior to posterosuperior as type C based on the classification of Eysel and Roosen [8], posterior stabilization C1/2 regarding to Goel/Harms [5, 7] was performed. This technique was also used in cases of pseudarthroses, pathological fractures or rheumatoid instabilities of C1/2.

Excluded were patients aged younger than 18 years as well as patients where multilevel stabilizations were needed.

### Operative treatment

#### Anterior procedures [lag screws, combined anterior odontoid and transarticular C1/2 screw fixation (AOTAF)]

The operations and surgical procedures were performed in supine position on a carbon table. An endotracheal anesthesia was done. Initially, in case of fractures closed reduction by traction and reclination was performed under fluoroscopic control if necessary. The patient’s head was placed in an adjusted padded carbon occipital ring. A right-sided anteromedial approach of 5–6 cm length was used in all cases. The further approach and the screw fixation of the odontoid process were done analogous to Etter et al. [9]. Due to lack of space, only one screw was placed into the odontoid process in some of the patients. In case of AOTAF bilateral atlantoaxial lateral mass screws were placed. The entrance points of those screws were right lateral to the odontoid screws medial of the sulci described by Lu et al. [14] and Reindl et al. [2] in contrast to the technique described by Polli et al. [3]. Initially, thin 2 mm Kirschner wires (K-wires) were used. Under fluoroscopic control the entrance point was evaluated about 3 mm lateral to the odontoid midline. The direction was tilted 30°–40° dorsally in the sagittal plane and 20°–25° laterally in the frontal plane. For this purpose the vertebral artery had to be checked preoperatively. Hereafter the 2 mm K-wires were exchanged against thinner 1.2 mm K-wires for better image quality. 2D-fluoroscopic images in a.p. and lateral view were taken and evaluated. Then, the 3D-Scan was performed in an apnoe phase. On the one hand the image quality was evaluated like described below, on the other hand the accuracy of the placed K-wires were checked regarding misplacements. If the position was correct, the cannulated screws (3, 5 mm) were implanted; in case of misplacements a revision of the position was performed.

### Posterior stabilization C1/2 regarding Goel/Harms

Following sterile draping, a midline incision was performed from the occiput to the tip of the processus spinosus of C3.

For the insertion of the C1 lateral mass screws, the anatomical landmarks are on the same vertical line as the C2 pedicle lines just below the posterior lamina of C1 and above the C1/2 joint. The lateral mass of C1 is identified above the C1/2 joint. With the 2.0-mm drill guide with oscillating attachment and protective sleeve, the correct direction was identified. The drill was oriented parallel to the posterior C1 arch toward the anterior aspect of C1, in general with 10°–20° ascending direction whereas in the axial plane the drill is oriented 10° toward the midline.

For screw placement in C2, the dissection is carried out over the superior surface of the C2 pars interarticularis. With a small nerve hook or elevator, the medial part of C2 pars interarticularis is identified. The entry point is halfway between the upper and lower articular surface of C2 at the vertical line while bisecting the articular mass. Finally, the drill is oriented 25° upward and 15°–25° medially as checked clinically by palpation of the medial wall of the isthmus of C2 with lateral fluoroscopic control [5, 7].

After the drill canal is prepared, thin 1.2 mm Kirschner wires were put into the canal and the 2D-fluoroscopic images in a.p. and lateral view were taken and evaluated (Image 1). Then, the 3D-scan was performed and the results were evaluated like described below. If the position was correct, the cannulated screws (3, 5 mm) were implanted; in case of misplacements a revision of the position was performed.

Seven experienced surgeons were involved. All of them are certificated spine surgeons.

### 3D-set up

The 3D image intensifier, Ziehm Vision FD Vario 3D^©^, (Ziehm Imaging GmbH, Nürnberg, Germany) is positioned on the opposite side, the patient lies on a carbon table; the head is positioned in a carbon head frame.

The scan consisted of 110 single images with a radius of 136° and took 110 s in an apnoe phase of the patient. The obtained 3D-scan with multiplanar reconstructions and an isocentrically acquired image series were controlled and evaluated before implanting the screws.

### Image quality

The evaluation of the image quality and diagnostic accuracy was performed using a Dicom viewer (efilm, Merge eMed, Milwaukee, WI) by two independent orthopedic trauma surgeons (UJAS, JSJ; both consultants). The inter-rater reliability was calculated regarding to Cohen’s kappa statistics.

A modified numeric analogue scale (NAS) and point system were used to assess the image quality. The NAS system was used to determine an exact score for an image with 10 for excellent image quality and 0 for artifact or not useable, respectively. Furthermore, the point system regarding to Stübig et al. [15] (Table 1) was used to grade the image quality and clinical applicability.

**Table 1:** Point System regarding to Stübig et al. [15]

Points	Subjective image quality total	Delineation of cortical bone	Deliniation of articular surfaces	Artifacts	Clinical assessment total
1	Excellent quality	Excellent deliniation	Excellentdeliniation	No relevant artifacts	Very good evaluation, no open questions
2	Good quality	Acceptable deliniation	Good deliniation	Few artifacts, barely disturbing	Good evaluation despite minor quality defects
3	Acceptable quality	Acceptable deliniation	Acceptable deliniation	Moderate artifacts, slightly disturbing	Evaluation generally possible with some open questions
4	Somewhat reduced quality	Barely visible, blurred edges	Barely visible, blurred edges	Disturbing, evaluation rather limited	Limited evaluation, control scan recommended
5	Reduced quality	Completely blurred, no delineatin	Completely blurred, no deliniation	Very disturbing, evaluation impossible	No evaluation of query, CT recommended

### Wire position

The position of all wires was analyzed.

Criteria for a misplaced wire were pedicle penetration of the wires medial, lateral or anterior, furthermore a wire placement not sufficiently positioned in the C1 mass and penetration of the anterior cortical edge of the C1 lateral mass. The criteria were stated before the study.

Based on the experience of the authors, the wires in the dens axis can be evaluated sufficiently under 2D fluoroscopy. This was not always the case in the wires or screws of C1 und C2 and the atlantoaxial screws.

Thus, malposition requiring reposition was defined as a clear pedicle penetration of the wires or wire placement not sufficiently positioned in the C1 mass. In these cases, the wires were removed and re-placement was performed.

In addition, in the authors experience the violence of the atlantooccipital joint by wires and screws cannot sufficiently be seen under fluoroscopic control. Thereby, in cases of wire violation of the atlantooccipital joint, the wires were slightly withdrawn backwards before screw length was measured. Here, no correction in terms of a new placement had to be done.

### Indications for additive intraoperative 3D Scan or postoperative CT

In case of malposition another 3D-scan was performed after the screws were implanted. If the wire-position was defined as correct, no additional 3D-scan was performed. Regarding to our institution standard a postoperative CT is only performed in case of neurological complications or problems regarding the respiration. In the patients of the current study no complication occurred.

### Statistics

All data underwent statistical analysis using standardized SPSS software 17.0 (SPSS, Inc. Chicago, USA). Statistical analysis was made using descriptive statistics. Fisher’s exact test was used to evaluate any associations between image quality and artifacts. A significance level of 0.05 was used.

## Results

### Patient collective

In 58 patients (33f, 25 m, average age 75.2 years, r.:18–95) with pathologies of the dens axis (45 type II fractures according to Anderson/D'Alonzo with or without arthrosis of C1/2, 2 unhappy triad of C1/2 (Odontoid fracture Type II, anterior or posterior C1 arch-fracture, Arthrosis C1/2) 4 pathological fractures, 3 pseudarthroses, 3 instabilities of C1/2 because of rheumatoid arthritis, 1 C2 arch fracture) 36 patients were treated from anterior [29 AOTAF (combined anterior odontoid and transarticular C1/2 screw fixation), 6 lag screws, 1 cement augmented lag screw] (Table 2) and 22 patients from posterior (stabilization C1/2 regarding to Goel/Harms [5, 7], Table 3).

**Table 2:** Patient population with treatment via anterior approach

n	Age	Gender	Diagnosis	Duration 3D Scan (s)	Dental implants	Image quality NAS	Malposition	Malposition correction
1	61	m	And Type II	242	Yes	7	No	No
2	90	m	And Type II + OA	258	No	9	C1 leftside too medial	Yes
3	89	f	And Type II + OA	237	Yes	6	No	No
4	28	m	And Type II	287	No	10	No	No
5	78	f	And Type II	241	No	9	No	No
6	80	m	And Type II + OA	255	No	9	No	No
7	88	f	And Type II	258	No	9	No	No
8	84	f	And Type II + OA	242	No	9	No	No
9	86	f	Unhappy triad	232	No	8	No	No
10	88	f	And Type II + OA	237	No	9	C2 leftside too long	draw back wire
11	95	f	And Type II + OA	259	No	9	No	No
12	76	f	And Type II + OA	247	Yes	7	No	No
13	80	m	And Type II + OA	287	Yes	8	C2 leftside too lateral	Yes
14	75	m	And Type II	299	Yes	7	No	No
15	88	f	And Type II + OA	239	No	9	C2 too long	draw back wire
16	92	f	And Type II + OA	254	No	9	No	No
17	90	f	And Type II + OA	281	Yes	7	No	No
18	76	m	And Type II + OA	240	Yes	7	No	No
19	77	m	And Type II + OA	288	Yes	7	No	No
20	86	f	And Type II + OA	252	No	8	C1 leftside too medial	Yes
21	78	f	And Type II + OA	263	Yes	9	No	No
22	18	m	And Type II	251	No	9	No	No
23	89	m	And Type II + OA	290	No	9	No	No
24	83	m	And Type II + OA	310	Yes	8	No	No
25	79	f	And Type II + OA	237	No	10	No	No
26	67	m	And Type II	285	Yes	8	No	No
27	92	f	And Type II	266	Yes	7	No	No
28	79	f	And Type II + OA	252	No	10	No	No
29	79	f	And Type II + OA	258	No	10	C2 too medial	Yes
30	78	m	And Type II + OA	248	Yes	7	No	No
31	83	m	And Type II + OA	272	No	9	C2 too long	draw back wire
32	75	f	And Type II + OA	261	Yes	8	No	No
33	93	f	And Type II + OA	281	No	8	C2 too long	draw back wire
34	83	f	And Type II + OA	309	No	9	No	No
35	84	m	And Type II + OA	242	Yes	7	No	No
36	87	f	And Type II + OA	257	No	10	C1 rightside too cranial	Yes

**Table 3:** Patient population with treatment via posterior approach (all treated with posterior stabilization C1/2 regarding Goel/Harms [5, 7]

n	Age	Gender	Diagnosis	Duration 3D Scan (s)	Dental implants	Image quality NAS	Malposition	Malposition correction
1	88	m	And Type II Eysel/Roosen Type C	257	No	10	No	No
2	69	m	Pathologic fracture	232	Yes	7	C1 too cranial	Yes
3	69	m	And Type II Eysel/Roosen Type C	301	No	8	No	No
4	79	f	And Type II Eysel/Roosen Type C	243	No	9	C2 rightside too medial	Yes
5	68	f	Pseudarthrosis	307	No	9	No	No
6	48	f	Instability C1/2	255	No	8	No	No
7	87	f	Unhappy triad	246	Yes	8	No	No
8	59	m	Instability C1/2	288	Yes	7	No	No
9	64	m	And Type II Eysel/Roosen Type C	255	No	8	No	No
10	71	m	Pathologic fracture	307	No	9	No	No
11	64	m	Instability C1/2	289	Yes	7	C2 leftside too medial	Yes
12	55	f	And Type II Eysel/Roosen Type C	247	Yes	8	No	No
13	53	m	And Type II Eysel/Roosen Type C	255	No	8	C2 too long	draw back wire
14	77	f	Pathologic fracture	279	Yes	7	C1 too long and too medial	Yes
15	83	m	Pseudarthrosis	252	Yes	7	No	draw back wire
16	81	f	C2 Arch fracture	288	Yes	7	No	No
17	53	f	And Type II Eysel/Roosen Type C	271	No	9	No	No
18	78	m	Pathologic fracture	243	No	9	No	No
19	80	f	And Type II Eysel/Roosen Type C	281	Yes	8	C1 not enough convergence	Yes
20	25	m	And Type II Eysel/Roosen Type C	258	No	9	No	No
21	85	f	And Type II Eysel/Roosen Type C	277	Yes	8	No	No
22	71	m	Pseudarthrosis	244	Yes	7	No	No

The implementation of the intraoperative 3D-Scan (preparing until image evaluation) took averagely 267 s (r.: 232–310 s). No technical problems occurred.

### Image quality

**Fig. 1:**
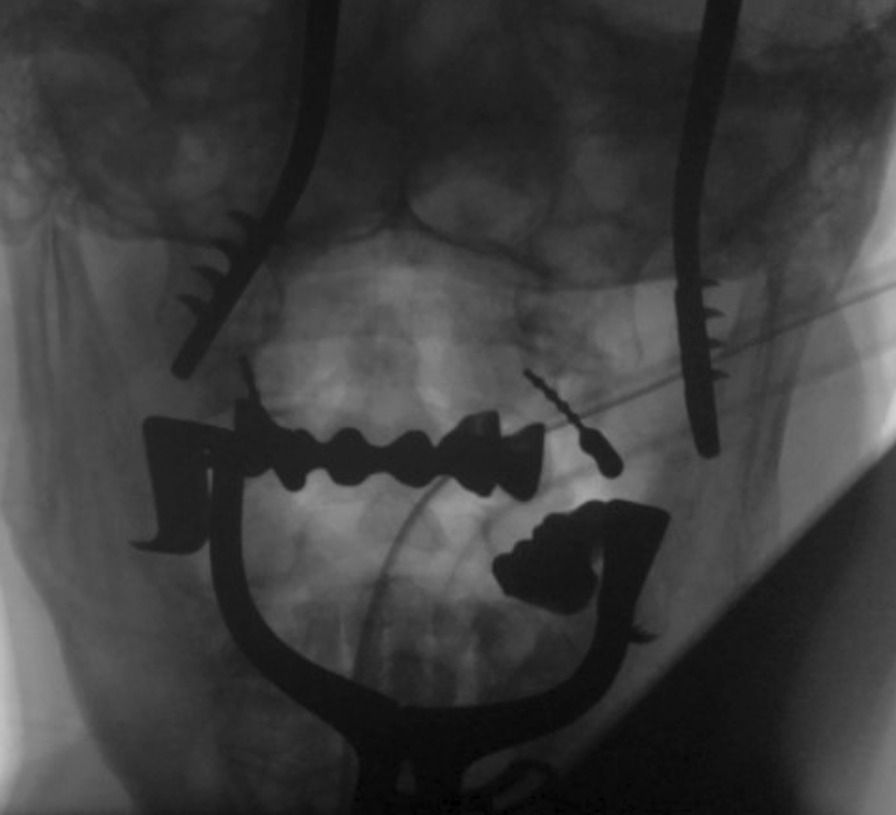
Example of a 2D fluoroscopy with K-Wires in C1

Regarding the NAS in the majority of the cases a very good image quality could be achieved. The median was 8,2 (r.: 6–10). In 41 (70.79%) patients the image quality was 8 or higher, in 17 Patients the image quality was deteriorated (NAS 7 = 16; 27.6%, NAS 6 = 1, 1,7%), all of these patients had dental implants (Table 2). The inter-rater reliability (kappa = 0.79) and the intra-rater (kappa = 0.77) reliability was high. In the point system regarding to Stübig et al. [15] 44.4% of the patients showed excellent quality concerning the subjective image quality, 23.4% good and 32.2% acceptable quality, the median was 1,88 (r.1–4). Concerning the delineation of the cortical bone 15 patients (25.9%) showed an excellent delineation of cortical bone, 26 (44.8%) good and 17 (29.3%) an acceptable delineation, the median was 2,1 (r.:1–3). The articular surfaces could be delineated excellent in 17 patients (29.3%), good in 23 (39.7%), acceptable in 17 patients (29.3%) and barely visible in 1 case (1.7%); the median was 2.1 (r.:1–4). Regarding the artifacts in 10 patients (17.2%) no relevant artifacts were visible. In 30 patients (51.7%) showed few artifacts, in 17 patients (29.3%) moderate artifacts were slightly disturbing. In one case (1.7%) the artifacts were disturbing with a rather limited evaluation. Overall, the median was 2,2 (r.:1–4). Concerning the total clinical assessment 10 patients (17.2%) were evaluated as very good with no open questions, 33 patients (56.9%) were good to evaluate with despite minor quality effects. Finally, 15 patients (25,9%) showed a general possible evaluation. The median was 2,11 (r.: 1–3) Overall, we found a significant reduction of image quality in patients with dental implants. Nevertheless, a reliable assessment of the K-wire placement could be realized in all cases (Fig. 1).

### Wire position

**Fig. 2:**
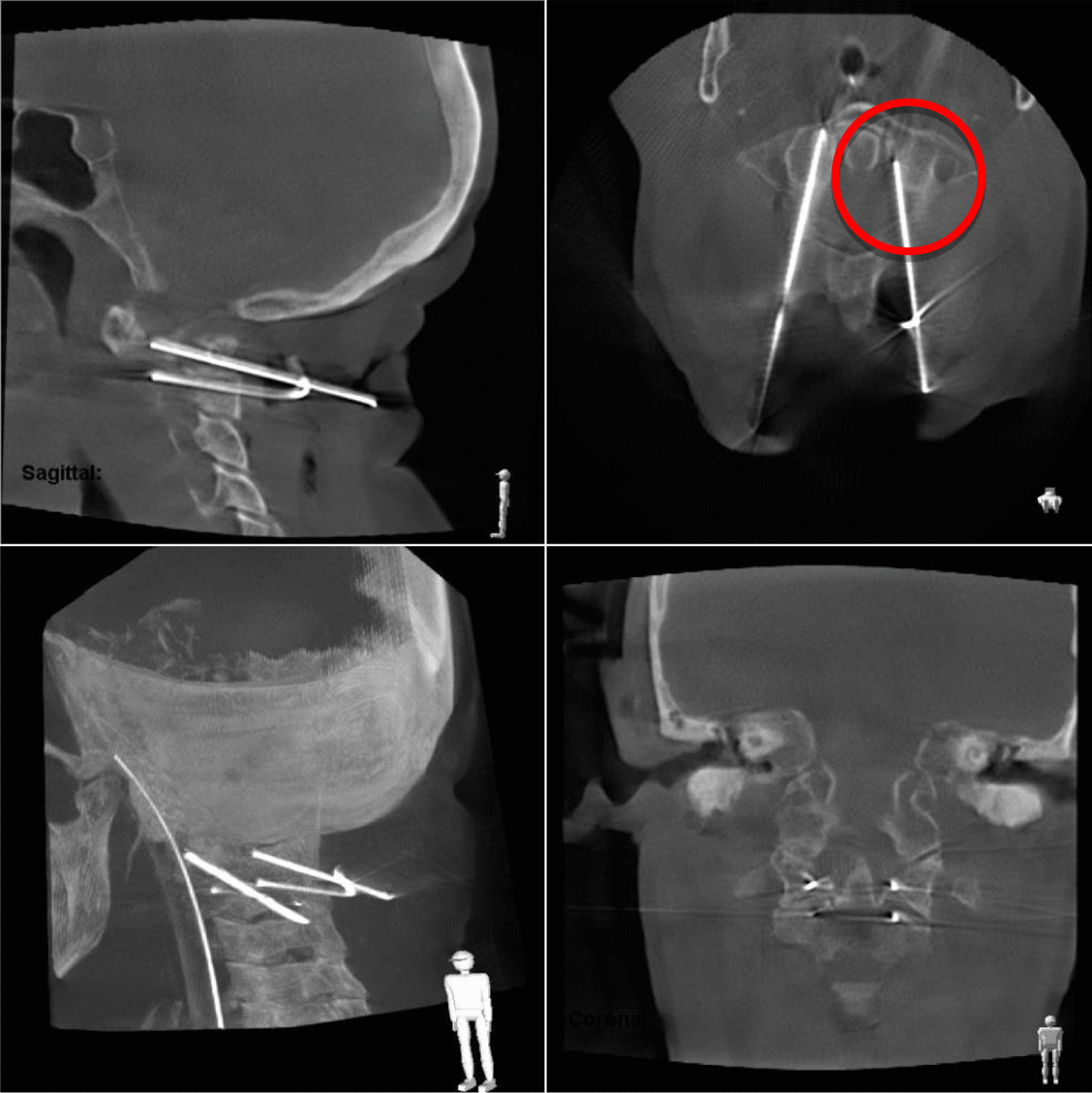
Example for misplacement of K-wire, in this case too medial in C1 left side

Out of a total of 148 intraoperatively controlled K-wires, 133 (89.9%) showed a correct positioning. In the other 15 (10.1%) cases a repositioning had to be done (n = 8; 5.4%) or it had to be drawn back (n = 7; 4.7%). A second 3D scan was performed in those 8 patients in which a malposition of the wires was visible and a new drill channel had to be generated. In the additional 7 patients no additional 3D-scan was performed. As the wires only had to be drawn back. A repositioning was possible in all cases (Fig. 2).

## Discussion

The operative treatment of pathologies in the upper cervical spine remains a complex procedure. Based on postoperative CTs high frequencies up to 21% of screw breaches through the cortex of the C2 pedicle or C1 lateral are seen, underlining a potential danger to nerves and blood vessels, especially the vertebral arteries and the spinal cord [10, 15]*.* Jacobs et al. [16] demonstrated that narrow pedicles lead to a higher perforation rate of the pedicle wall. According to their study pedicles of < 6.6 mm turned out to be a risk factor for a perforation of the pedicle wall.

Apart from that, malposition is associated with a potential loss of stability. As only thin corridors exist for the implementation of screws, several drill attempts also can lead to screw dislocations as seen in a recent study to AOTAF [4].

To improve accuracy and safety, computer-aided 3-dimensional (3D) imaging is widely used and has proven to be effective [4, 14, 17–21]*.* It provides better image quality for the anatomical landmarks intraoperatively, especially the complex atlantoaxial region, compared with conventional fluoroscopy [22]. Moreover, instrumentation in critical anatomical regions can already be controlled intraoperatively. The important role of intraoperative multiplanar imaging after osteosynthesis is supported by the high rate of immediate corrections for 11–39% of other anatomic regions in the literature [18–21, 23–25]. Hence, the additional medical benefit seems undisputed [13, 26]*.*

In this study we proved the applicability and advantages of intraoperative imaging using a 3D flat panel in the treatment of C1/2 pathologies. Instead of controlling the final placement of the screws we scanned the trajectory of the drilling canal in term of the K-wires, which were put into the thin drilling canal. Hence a possible correction after a scan could be easily performed. This might be more difficult when a screw with a thicker diameter has already been implanted. Therfore we tactically moved one step forward regarding the 3D-Scan. In the present study the huge majority, (133, 90%), out of a total of 148 intraoperatively controlled K-wires, were placed correctly and only 10% required repositioning due to not optimal positioning. Repositioning was possible in all cases intraoperatively. Thereby, insufficient screw hold or drastic complications such as neurologic deficits or bleeding could be avoided.

Concerning the image quality our results demonstrated that the average score for the overall image quality was about 8 with the NAS, and mainly very good or good for the point system. The clinical assessment score was good despite minor quality defects. Artifacts mainly were seen when the patients had dental implants. Here, a significant correlation could be found. Nevertheless, misplacements also occurred in cases of good or very good image quality and independently of dental implants, here no significant correlation could be found.

Apart from that we have not seen other factors influencing the image quality such as osteoporosis or obesity [27]. In other parts of the human body like the upper thoracic spine or the pelvis region the image quality can be inferior because of the relatively thick soft-tissue envelope. A scan can also be difficult in obese patients in terms of realizing the rotation of the C-Arm. However, this is no problem in the area of the upper cervical spine.

Compared to other studies we can see an improvement in the image quality over the years. Although it is difficult to find comparable studies dealing with the image quality of 3D-scan of the upper cervical spine, former studies showed worse image qualities. Stübig et al. [15] received an average score for the overall image quality with about 7 with the NAS, and good or acceptable for the point system. The clinical assessment score was good despite minor quality defects.

Another often-discussed issue is the increased time required by the use of intraoperative 3D-image devices. In our study, however, the implementation of the intraoperative 3D-scan (from preparing of the C-Arm until image evaluation) took averagely only 267 s (r.: 232–310 s). We did not face any technical problems. Here we can see a significant improvement over the previous generations of 3D-C-Arms, where technical problems were more common and the duration before the surgeon could start to evaluate the images was longer.

This prolonged operation time also can be seen in combination with navigated procedures. In a former study of the authors the average time for a scan including the preparation was 409 s [1]; in addition we faced technical problems in several cases concerning the combination of 3D-C-Arm and navigation system. Apart from that a prolongation has to be taken into account especially at the beginning of using navigation tools due to the learning curve [28]. Furthermore, accuracy problems can occur in case of instable situations in the upper cervical spine. The reference clamp has to be fixed securely to avoid malposition guided by a false navigation.

Several studies demonstrate the safety of navigated odontoid screw placement using O-arm precursors and suggest possible better outcomes compared with conventional techniques. The majority of these studies have compared fluoroscopy-based techniques with the Iso-C system [26]. For instance, Martirosyan et al. [11] compared biplanar fluoroscopy–guided anterior screw fixation of odontoid fractures in 25 patients with Iso-C-guided anterior screw fixation in 26 patients. Surgical complication rates and clinical outcomes were also similar between the groups.

Martirosyan et al. [11] did not detect significant differences in complication rates between patients in whom the O-arm was used and those in whom it was not used as an adjunct. Similar to other 3D-imaging-assisted studies of anterior odontoid fixation, no screws required immediate replacement.

Regarding to the literature the use of intraoperative 3D-Devices with navigation for stabilizations of the upper cervical is technically feasible and shows an increased accuracy of screw placement [13, 18, 29]. Other current alternatives for safe screw implantation are 3D-printed patient specific templates. Tian et al. [30] compared templates with C-Arm navigation and achieved more accurate C2 pedicle screw placement with the 3D-printed navigation template technology although both 3D-printed navigation template-assisted and C-arm based navigation-assisted C2 pedicle and pars screw placement provided similar safety and clinical efficacy. One disadvantage of the templates is the time needed for the production; therefore urgent cases cannot be treated with these devices. Another problem might be the high costs, which also is one disadvantage of navigation systems. This issue is often used as an argument against 3D-C-Arms, possibly connected with other oblique purchases, for example, a carbon operation table or a carbon head holder, as metal artefacts affect the image quality quite seriously. Hüfner et al. [17] highlighted this topic and concluded that, even though the price of a 3D unit is high, the long-term perioperative costs could be decreased by frequent application of this device and, thereby, higher rates of avoided revisions. However, a substantial deficit could arise if the revision rate is only up to 5%.

## Limitations

Our study offers some limitations. Even if a wire is placed correctly, the screw still can be misplaced, especially in osteoporotic bone. Apart from that, the initial placement of the wires or the drilling process can also lead to relevant complications. Here, navigated procedures could bring more safety. Another drawback is the fact that no additive scan was performed after implanting of the screws or a revision regularly. We only perform a second scan in cases of insecure evaluation of the implants, which was not necessary in our study; nevertheless we did not see screw-related complication postoperatively. Apart from that there was no control group, so that we are not in the position to demonstrate the clinical advantage.

## Conclusion

Intraoperative 3D imaging in the upper cervical spine is fast and easy to perform with sufficient image quality even in patients with dental implants. A screw canal malposition can be detected by wire positioning in the drill cannel prior 3D scanning. If necessary, intraoperative correction was possible in all patients. Improved drill cannel position could be achieved based on the thin drill canal that was created compared to larger bony defects seen after inserting a definite screw.

## Data Availability

The datasets used and/or analysed during the current study are available in the additional material except identifying/confidential patient data.

## Abbreviations

AOTAF: Anterior screw osteosynthesis and anterior transarticular C1/2 screw fixation
AO: Atlanto-odontoid osteoarthritis
K-Wires: Kirschner wires
NAS: Numeric analogue scale
f: Female
m: Male
n: Number
r: Range
